# Avian Influenza A (H5N1) Virus Antibodies in Poultry Cullers, South Korea, 2003–2004

**DOI:** 10.3201/eid1806.111631

**Published:** 2012-06

**Authors:** Donghyok Kwon, Joo-Yeon Lee, Wooyoung Choi, Jang-Hoon Choi, Yoon-Seok Chung, Nam-Joo Lee, Hyang-Min Cheong, Jacqueline M. Katz, Hee-Bok Oh, Haewol Cho, Chun Kang

**Affiliations:** Korea Centers for Disease Control and Prevention, Osong, South Korea (D. Kwon, J.-Y. Lee, W. Choi, J.-H. Choi, Y.-S. Chung, N.-J. Lee, H.-M. Cheong, H.-B. Oh, H. Cho, C. Kang);; Centers for Disease Control and Prevention, Atlanta, Georgia, USA (J.M. Katz)

**Keywords:** influenza, H5N1, transmission, disease outbreaks, microneutralization, viruses, antibodies, avian, poultry, bird cullers, poultry cullers, South Korea

## Abstract

Transmission of influenza (H5N1) virus from birds to humans is a serious public health threat. In South Korea, serologic investigation among 2,512 poultry workers exposed during December 2003–March 2004 to poultry with confirmed or suspected influenza (H5N1) virus infection found antibodies in 9. Frequency of bird-to-human transmission was low.

The highly pathogenic avian influenza (H5N1) virus has posed a serious public health threat since 1997, when the first transmission of the virus from birds to humans was reported in Hong Kong (*1**,**2*). In South Korea, during December 2003–March 2004, this virus caused 19 outbreaks in 7 provinces (10 outbreaks on chicken farms and 9 on duck farms), which prompted a massive mobilization to cull birds and contain the outbreak (*3*). Vaccination of poultry against influenza (H5N1) virus was legally prohibited, and a stamping out policy was considered as a control option. Culling of ≈5 million birds was conducted on all farms with infected poultry and all poultry farms within a 3 km–radius protection zone.

All persons who participated in the culling operations were equipped with World Health Organization (WHO)–recommended personal protective equipment (PPE) (*4*). To prevent the possibility of mixed infection with human and avian influenza viruses, previously nonvaccinated participants were vaccinated with a seasonal influenza vaccine and given oseltamivir as an additional prophylactic measure.

During the outbreaks, 142 respiratory specimens were collected from persons who had influenza-like illness and tested by reverse transcription PCR selective for the matrix and hemagglutinin (H) 5 genes and by virus isolation in cell culture; however, no influenza (H5N1) virus was detected (Korea Centers for Disease Control and Prevention [CDC], unpub. data).

The definition of a case of influenza-like illness was sudden onset of fever (>38°C) with cough or sore throat. According to previous serosurveys and outbreak investigations, influenza (H5N1) virus is poorly transmitted from birds to humans (*5**–**8*). To trace the frequency of transmission of the influenza (H5N1) virus to persons who had been exposed to the confirmed or suspected virus-infected poultry, a serosurvey was conducted by the Korea CDC.

## The Study

A serologic investigation was performed among 2,512 persons who worked on poultry farms or culled birds during the 2003−2004 outbreaks in South Korea. Their use of PPE, receipt of oseltamivir, and exposure to birds with confirmed influenza (H5N1) is unclear. Poultry culling was conducted during December 12, 2003–March 21, 2004. Blood was collected from cullers on the day of culling completion in each region. Convalescent-phase blood samples were collected at least 4 weeks later. Written agreement was provided before blood was collected from cullers, other poultry workers, and their household members. This study was reviewed and approved by the ethics committee of Korea CDC.

WHO-recommended laboratory tests and case definitions were used for serologic diagnosis of influenza (H5N1) virus infection in the cohort (*9**–**11*). Before this study, the laboratory staff of Korea CDC received 4 weeks of training at the US CDC on serologic testing for influenza (H5N1) virus. All experiments with live viruses were conducted at the biosafety level-3 facility of Korea CDC, and all serologic testing at the US CDC was conducted under biosafety level-3 containment including enhancements required by the US Department of Agriculture and the Select Agent Program.

All serum samples were tested for antibodies against influenza (H5N1) virus by microneutralization (MN) assay; results were considered to be positive if titers against H5 were >80 according to at least 2 independent assays. As recommended by WHO, samples that were antibody-positive by MN underwent confirmatory testing by hemagglutination inhibition assay with horse erythrocytes or by H5-specific Western blot analysis (*9**,**10*).

During the 2003−2004 outbreaks, 4,108 serum samples were collected from 2,820 persons. However, ≈16% of the samples showed cytotoxicity (all cells were detached on the 96-well microplates after fixation with acetone) on MDCK cells during MN assay. In total, 3,448 samples from 2,512 persons were analyzed, among which paired samples were available from 936 (37%) and a single sample was available from 1,576 (63%). The median age of the participants was 36.0 years (range 3−96 years), and 2,112 (84.1%) were male. Among those for whom epidemiologic data were available, 1,327 (84.3%) were cullers and 176 (11.2%) were farm workers or their household members (Table 1). Cullers included local government workers, soldiers, animal husbandrymen, and civilians. The culling periods were 1–13 days, and the average was 5.4 days.

**Table 1:** Demographic characteristics of 2,512 bird cullers exposed during December 2003–March 2004 to poultry with confirmed or suspected influenza (H5N1) virus infection, South Korea

Characteristic	No. (%) cases	No. (%) positive
Age group, y, n = 2,055*		
<20	74 (3.6)	0
20–29	783 (38.1)	5 (0.58)
30–39	365 (17.8)	1 (0.27)
40–49	427 (20.8)	3 (0.70)
50–59	249 (12.1)	0
>59	157 (7.6)	0
Sex, n = 2,512		
M	2,112 (84.1)	9 (0.43)
F	400 (15.9)	0
Type of work, n = 1,573		
Poultry farm worker†	176 (11.2)	0
Culler	1,327 (84.3)	9 (0.68)
Other‡	70 (4.5)	0

*No. persons whose epidemiologic history was available. †Includes farm workers and their household members. ‡Includes epidemiologists, public health officials, and media reporters.

Among the 2,512 persons, MN assay results were confirmed positive for 9. The US CDC confirmed positive results in a single sample for 4 persons; the Korea CDC confirmed positive results in paired samples for 5 others. Among the 9 persons with positive MN results, only 2 had positive results according to horse hemagglutination inhibition assay; however, all 9 had clear reactivity to H5 proteins on Western blot analysis and were confirmed positive according to WHO criteria (Table 2). All those with influenza (H5N1)–positive results were male, median age was 32.5 years (range 22–48 years), and all had participated in culling during the outbreaks (Table 2). None of the other poultry farm workers had seropositive results.

**Table 2:** Characteristics and serologic results of persons with influenza (H5N1) antibody–positive serum samples, South Korea, 2003–2004*

Participant no.†	Age, y	Occupation	Serologic results‡		
			Neutralizing antibody titer	Horse HI titer	Western blot§
1	48	Fireman	<20/80	<10/80	−/+
2	23	Soldier	<20/80	<10/160	−/+
3	23	Soldier	20/640	<10/40	+/+
4	23	Soldier	20/160	<10/<10	+/+
5	25	Soldier	20/160	<10/<10	±/+
6	48	Provincial officer	160	<10	+
7	22	Soldier	80	<10	+
8	36	Provincial officer	80	160	+
9	45	Animal husbandryman	80	80	+

*All 9 persons were male, worked as cullers, and had no signs or symptoms of respiratory illness. HI, hemagglutination inhibition assay; +, positive; −, negative; ±, equivocal. †Cases 1–5 were confirmed at the Korea Centers for Disease Control and Prevention with paired samples and 6–9 were confirmed at the US Centers for Disease Control and Prevention with a single sample. ‡Results for participants 1–5 were obtained from 2 samples and for 6–9 from 1 sample. §Western blot analyses were performed by using purified baculovirus-expressed influenza A/Vietnam/1203/2004 virus hemagglutinin as antigen.

## Conclusions

By identifying only 9 seropositive cases among 2,512 persons, we determined that the risk for poultry-to-human transmission of the influenza (H5N1) virus is small. Other studies have also shown low frequencies of poultry-to-human (H5N1) virus transmission. In provinces in Thailand, blood samples were collected from 22 poultry farmers 6 months after confirmation of influenza (H5N1) virus outbreaks; all antibody titers were negative (*5*). Two studies of villagers in Cambodia who had frequent and direct contact with poultry with confirmed and suspected influenza (H5N1) virus infection found low frequency of virus transmission from poultry to humans (*6**,**7*). Similarly, a study in Nigeria also found negative results for antibodies against influenza (H5N1) virus among 295 poultry workers (*8*).

Because our study was conducted as a public health response, it has the following limitations. We were unable to systematically assess symptoms, extent of exposure, compliance with PPE use, and taking of oseltamivir. It is not clear if the participants were exposed to birds with confirmed or suspected influenza (H5N1) virus or whether they wore PPE properly when culling. Because the outbreak created an emergency situation and this study had not been designed before the outbreak, epidemiologic data were limited. And because we had insufficient serum for adsorption assays, we cannot exclude the possibility of cross-reactivity with circulating antibodies resulting from seasonal influenza vaccination or previous infection with human influenza virus. In 2004, among 83 Vietnam hospital employees who were exposed to 4 patients with confirmed and 1 patient with probable influenza (H5N1) virus infection, a positive antibody titer against influenza (H5N1) virus and cross-reacting antibodies against influenza (H1N1) virus was found on MN assay for 1 employee (*12*). Because our study was not a case–control study, we could not identify risk factors for transmission.

Regardless of these limitations, our study shows serologic evidence of influenza (H5N1) virus transmission among groups at high risk for poultry-to-human transmission (i.e., exposed to poultry during 2003−2004 outbreaks in South Korea). However, we also found additional proof that the frequency of poultry-to-human influenza (H5N1) virus transmission is low.
